# Literary Notes

**Published:** 1909-07-24

**Authors:** 


### Literary Notes.

In a recent review in these columns of one of the Oxford Medical Publications it was stated that the variety of *format* adopted in this series necessitated the volumes being separated on the purchaser's bookshelves. With the doctor's, as with every private library, indeed, there is always the question as to whether the arrangement of the books shall follow size or subject. The former plan has good precedents : in the top rows the owner's eye can find affectionately, as Mr. Austin Dobson sings, "the dear and dumpy twelves"; while nearer the floor, as is fitting, come the weightier volumes—like those Charles Lamb wished to strip in order to clothe his favourites better. The second arrangement, more convenient for frequent reference, has obvious drawbacks, especially in the case of periodical medical literature, a very varied selection of which is apt to be made by those who join to the investigator's insatiable curiosity concerning his own subject something of the pride of the collector in completeness. *Virchow's Archiv*, say, with its mere eight by six dimensions, makes but a poor prop for some limp, lanky reprint of a Royal Society memoir; and yet the two may often need to be consulted together. Indeed, there is little to charm the bibliophile in the appearance of a useful small medical library.

### THE LEEDS HEALTH CONGRESS.

A Health Congress, organised by the City and University of Leeds, with the co-operation of the Royal Sanitary Institute and the Royal Institute of Public Health, was opened at Leeds on Saturday evening last, and continues open throughout the present week. Over 1,000 delegates, representing nearly every branch of medical and sanitary science, were received at the University by the Lord Mayor (Alderman F. J. Kitson) and Lady Mayoress of Leeds.

The Presidential address was delivered by Colonel T. W. Harding, of Leeds. He said that division of labour in scientific matters whereby individual workers applied themselves to the investigation of some detail or group of details, whilst possessing great advantages, also was associated with a certain degree of danger. The limited vision of the specific worker might obscure wider issues and even disturb his sense of proportion of the particular parts he was considering. But while he appreciated the great progress of sanitary science, he doubted whether we were healthier than were our fathers, because we had lost some conditions that were simple, rational, and good. The population in their day was small; the great bulk of the people worked in the fields and rose with the sun; the woman's place was in her home; the children were breast-fed and brought up at their mother's knee; their education was of the simplest, mostly in the church, and the boys were content to walk in their fathers' footsteps. The striking difference in the old conditions as compared with the new was that in former days 75 per cent. of the population was rural and only 25 per cent. urban, while now 77 per cent. was urban and only 23 per cent. rural. Phthisis was being brought under control by administrative and educational measures, but still had a terrible mortality. The birth-rate had declined since 1880. Some of the influences of this were obscure, but they were not all vicious, and in so far as early, improvident marriages had been reduced, it had been to the public and private advantage. He warmly approved of physical exercises in schools, and he hoped to see military drill and discipline for boys followed later by universal military training.

THE HOSPITAL July 24, 1909.

